# Necroptosis in Macrophage Foam Cells Promotes Fat Graft Fibrosis in Mice

**DOI:** 10.3389/fcell.2021.651360

**Published:** 2021-03-25

**Authors:** Xihang Chen, Zilong Deng, Jingwei Feng, Qiang Chang, Feng Lu, Yi Yuan

**Affiliations:** ^1^Department of Plastic and Cosmetic Surgery, Nanfang Hospital, Southern Medical University, Guangzhou, China; ^2^Department of Stomatology, Nanfang Hospital, Southern Medical University, Guangzhou, China

**Keywords:** fat grafting, fibrosis, macrophage foam cells, necroptosis, fibroblast

## Abstract

**Background:** Fibrosis is a major grafting-related complication that leads to fat tissue dysfunction. Macrophage-induced inflammation is related to the development of fat tissue fibrosis. Necroptosis is a recently discovered pathway of programmed cell necrosis that results in severe inflammation and subsequent tissue fibrosis. Thus, in this study, we investigated the role of macrophage necroptosis in fat graft fibrosis and the underlying mechanisms.

**Methods:** Fibrosis and necroptosis were investigated in mouse fat tissue before and after grafting. An *in vitro* “crown-like” structure (CLS) cell culture model was developed by co-culturing RAW 264.7 macrophages with apoptotic adipocytes to reproduce *in vivo* CLS macrophage-adipocyte interactions. Lipid uptake and necroptosis in CLS macrophages were analyzed using Oil-Red-O staining, western blotting, and immunofluorescence. RAW264.7 macrophages were cultured alone or with apoptotic adipocytes and treated with a necroptosis inhibitor (Nec-1 or GSK872) to explore the paracrine effect of necroptotic CLS macrophages on collagen synthesis in fibroblasts *in vitro*. Mice were treated with Nec-1 to analyze the effect of blocking necroptosis on fat graft fibrosis.

**Results:** Fibrosis was increased after grafting in fat grafts of mice. Macrophages clustered around apoptotic adipocytes or large oil droplets to form a typical CLS in fibrotic depots. This was accompanied by formation and necroptosis of macrophage foam cells (MFCs) in CLSs. RAW 264.7 macrophages co-cultured with apoptotic adipocytes induced CLS formation *in vitro*, and lipid accumulation in CLS macrophages resulted in the formation and necroptosis of MFCs. Necroptosis of MFCs altered the expression of collagen I and VI in fibroblasts via a paracrine mechanism involving inflammatory cytokines/chemokines, which was reversed by GSK872 or Nec-1 treatment. Furthermore, treatment with Nec-1 ameliorated fat graft fibrosis in mice.

**Conclusion:** Apoptotic adipocytes induced necroptosis of MFCs, and necroptosis of these cells activated collagen synthesis in fibroblasts via a paracrine mechanism. Inhibition of necroptosis in macrophages is a potential approach to prevent fibrosis in fat grafts.

## Introduction

Fat grafting is a promising regenerative cell-directed therapy that has shown good results in several applications such as breast augmentation and reconstruction, treatment of contour deformities and scars, and wound healing (Klinger et al., 2008; Slack et al., 2014; Condé-Green et al., 2015; Katzel and Bucky, 2017; Ruan et al., 2019). However, fat graft fibrosis is a major grafting-related complication (Mineda et al., 2014; Cai et al., 2017). Fibrosis is characterized by accumulation of disorganized and stiff extracellular matrix (ECM). ECM deposition is linked to fat tissue dysfunction (Guglielmi et al., 2015; Marcelin et al., 2017). Targeting ECM remodeling improves the metabolic function of fat tissue (Vila et al., 2014). However, the molecular mechanisms underlying fat graft fibrosis are largely unknown. Aberrant inflammation contributes to tissue fibrosis (Tanaka et al., 2014). Macrophages are involved in fat graft inflammation via interactions with adipocytes. Adipocytes in fat grafts die soon after grafting, which may cause lipid spillage from adipocytes, resulting in lipid toxicity, and subsequent inflammation (Eto et al., 2012). Macrophages clear apoptotic/dead adipocytes by engulfing their remains, which mainly consist of large lipid droplets (Mashiko and Yoshimura, 2015), via an efferocytosis process (Boada-Romero et al., 2020). This leads to the formation of lipid-laden macrophages known as macrophage foam cells (MFCs; Webb and Moore, 2007; Hagit et al., 2013). MFCs cause severe inflammatory changes during fat tissue remodeling in obesity (Haka et al., 2016; Coats et al., 2017). However, whether MFCs are present and affect inflammation in fat tissue, thereby regulating tissue remodeling after grafting, and the underlying mechanisms remain unclear.

Aberrant accumulation of lipid droplets may impair the degradative capacity of macrophages and cause cell lipotoxicity (Sergin et al., 2015; Evans et al., 2018). In addition, dysregulated lipid metabolism can alter macrophage activation and cause necrosis, which may be accompanied by uncontrolled release of inflammatory mediators, proteases, and coagulation factors (Thorp et al., 2011; Tian et al., 2016). This leads to a hyperinflammatory state and dramatically induces tissue dysfunction, including abnormal tissue remodeling (Schott et al., 2019). Thus, lipotoxicity-mediated cell death might be a detrimental fate of MFCs related to graft tissue fibrosis.

The recent discovery and characterization of a programmed necrosis pathway, called necroptosis, expanded understanding of the mechanisms leading to cell death (Vandenabeele et al., 2010). Necroptosis is an endogenous trigger of inflammation that leads to tissue fibrosis (Choi et al., 2015; Xiao et al., 2017). It is mediated by the necrosome, a signaling protein complex composed of receptor-interacting protein kinases 1 and 3 (RIPK1 and RIPK3) and mixed lineage kinase domain-like (MLKL). Phosphorylated RIPK3 recruits and activates MLKL, which translocates to the cell membrane and causes its rupture. Owing to loss of membrane integrity in necroptotic cells, the intracellular contents act as damage-associated molecular patterns (DAMPs), which cause sterile inflammation in affected tissues (Kaczmarek et al., 2013). Necroptosis also promotes cell-autonomous production of proinflammatory cytokines (Zhu et al., 2018). Therefore, necroptosis is considered to be a highly inflammatory form of cell death.

Thus, we hypothesized that MFCs may undergo necroptosis in fat tissue after grafting, leading to hyperinflammation and fibrosis. We first found that the necroptosis pathway was triggered in MFCs located in fibrotic regions of fat grafts, and demonstrated that necroptosis of these cells activated collagen synthesis in fibroblasts via a paracrine mechanism *in vitro* and *in vivo.* Furthermore, we demonstrated that inhibition of necroptosis is a potential approach to prevent or treat fibrosis in fat grafts.

## Materials and Methods

### Animals

All experiments were approved by the Nanfang Hospital Animal Ethics Committee Laboratory and were conducted according to the guidelines of the National Health and Medical Research Council of China. C57/BL6 male mice were obtained from Southern Medical University.

### Fat Grafting Model and Treatments

Mice were anesthetized by intraperitoneal injection of pentobarbital sodium (50 mg/kg). Fat tissue was harvested from the inguinal fat pads of mice and gently dissected into very small pieces, similar to the size of aspirated fat tissue used for clinical fat injection in humans. The volume baseline for fat grafts was 0.3 mL of prepared adipose tissue. The recipient sites were the sides of the lower back. Mice were sacrificed after 1, 4, 8, 12, and 16 weeks (*n* = 5 per time point per group), the grafts were harvested and carefully separated from surrounding tissue, and their volumes were measured.

To investigate the effects of necroptosis inhibition *in vivo*, fat-grafted mice were intravenously injected with 1.65 mg/kg necrostatin-1 (Nec-1; Sigma-Aldrich, St. Louis, MO, United States; Linkermann et al., 2013; Caccamo et al., 2017; Liang et al., 2019) once per day for 30 days from the fourth week after grafting. Control fat-grafted mice were intravenously injected with the same volume of DMSO diluted in saline solution.

### Terminal Deoxynucleotidyl Transferase-Mediated dUTP Nick End Labeling

Transferase-mediated dUTP nick end labeling (TUNEL) staining was performed with the TUNEL apoptosis kit direct (Beyotime Institute of Biotechnology, China) according to the manufacturer’s instructions. Fat tissues were fixed with neutral buffered formalin and embedded in paraffin. Paraffin-embedded fat tissue sections were rehydrated and treated with proteinase *K* solution for permeation. The slides were immersed in terminal deoxynucleotidyl transferase (TdT) labeling buffer. Then, samples were covered with anti-bromodeoxyridine (anti-BrdU) and incubated with streptavidin-horseradish peroxidase (HRP) solution. Diaminobenzidine (DAB) was used as the chromogen. Cells containing fragmented nuclear chromatin, a characteristic of apoptosis, exhibit a brown nuclear staining.

### Histological Analyses

Four micrometer-thick sections were stained with hematoxylin and eosin (H&E), Masson’s trichrome, and Sirius red. Fibrosis-positive areas were measured using ImageJ software (National Institutes of Health, Bethesda, MD, United States).

For immunohistochemical analyses, fat tissue sections were incubated with the following primary antibodies: rat anti-mouse F4/80 (1:100; Abcam, Cambridge, United Kingdom) and rabbit anti-mouse perilipin and anti-S100A4 (1:1000, Abcam), anti-collagen I (1:200, Abcam), anti-collagen VI (1:200, Abcam), and anti-pMLKL (1:100; Invitrogen, Carlsbad, CA, United States). Sections were imaged using a light microscope (BX51; Olympus, Tokyo, Japan). The number of cells exhibiting brown staining of the cytoplasm was counted at 400× original magnification. Collagen-positive areas and lysosome diameters were measured using ImageJ software.

For immunofluorescence analyses, fat tissue sections were incubated with the following primary antibodies: rabbit anti-mouse perilipin (1:100, Abcam), rat anti-mouse F4/80 (1:100, Abcam), rabbit anti-mouse adipophilin (1:100, Abcam), rat anti-mouse F4/80 (1:100, Abcam), rabbit anti-mouse pMLKL (1:100; Cell Signaling Technology, Beverly, MA, United States), mouse anti-mouse S100A4 (1:100, Abcam), and rabbit anti-mouse collagen I (1:100, Abcam). Images were captured using a fluorescence microscope (IX71FL, Olympus). The numbers of F4/80-positive/pMLKL-positive cells were counted at 400× original magnification.

For transmission electron microscopy (TEM) analyses, fat tissue samples (1 mm × 1 mm × 1 mm) were further fixed overnight at 4°C in the same buffer. For post-fixation, tissue fragments were transferred to 1% osmium tetroxide and 1.5% potassium ferricyanide for 1.5 h. Then, samples were dehydrated with different concentrations of ethanol and acetone, embedded in epoxy resin, and sectioned at a thickness of 80 nm. After staining with uranyl acetate and lead citrate, ultrathin sections were imaged using a transmission electron microscope (Hitachi, Tokyo, Japan).

### Apoptosis of Adipocytes *in vitro*

3T3-L1 cells were differentiated into adipocytes as described previously (Haka et al., 2016) and used in experiments after 15–20 days. To recapitulate the apoptosis of adipocytes *in vivo*, adipocytes were incubated with tumor necrosis factor (TNF)-α (10 ng/ml) for 24 h, after which the culture medium was replaced by normal culture medium. The rate of apoptosis was analyzed by flow cytometry after double staining with propidium iodide (PI, Solarbio, Beijing, China) and FITC-conjugated annexin V (AnxV).

To distinguish between different forms of adipocyte death (apoptosis and necroptosis) *in vitro*, adipocytes were co-cultured with TNF-α (10 ng/ml) in the presence or absence of the necroptosis inhibitor Nec-1 (20 μM; Selleck, Houston, TX, United States) or GSK872 (3 μM, Selleck) for 24 h. Adipocyte death was detected by PI staining.

### Analysis of MFC Formation and Necroptosis *in vitro*

To induce the formation of MFCs, RAW 264.7 mouse macrophages were incubated alone or with apoptotic adipocytes for 24 or 96 h. MFC formation in crown-like structures (CLSs) was quantified by Oil Red O staining (Cyagen, Guangzhou, China).

To determine the functional role of MFC necroptosis, macrophages were co-cultured with apoptotic adipocytes in the presence or absence of the necroptosis inhibitor [Nec-1 (20 μM; Selleck) or GSK872 (3 μM, Selleck)] for 96 h. Then, cell culture media were collected and added to the medium of L929 mouse fibroblasts to explore the paracrine effect of necroptotic MFCs on fibroblasts.

### Phagocytosis Assays *in vitro*

RAW 264.7 mouse macrophages were incubated with FITC-AnxV-stained apoptotic adipocytes at a ratio of 2:1 for 24 or 96 h. Fluorescence microscopy detected fragments of apoptotic adipocytes in macrophages, demonstrating that macrophages specifically phagocytosed apoptotic adipocytes. The proportion of macrophages containing ingested apoptotic cells was determined by flow cytometry. Cells were harvested and incubated with PE-conjugated F4/80 antibody to label macrophages, and the phagocytic ratio was determined by flow cytometry. FITC + F4/80 + cells were identified as macrophage cells that ingested FITC-AnxV-stained apoptotic adipocytes.

### Hoechst 33342/PI Staining

Cells were stained with Hoechst 33342 (5 μg/mL) and PI (5 μg/mL; Solarbio) and imaged using a fluorescence microscope (Olympus). Necrotic cells were identified as those with PI-stained nuclei.

### Real-Time Polymerase Chain Reaction Analysis

Isolated and quantified RNA was used to synthesize cDNA using PrimeScript^TM^ RT Master Mix (TaKaRa, Kyoto, Japan). Polymerase chain reaction (PCR) was performed using a LightCycler 480 Real-time PCR System (Roche, Indianapolis, IN, United States) and SYBR^®^ Premix Ex Taq^TM^ (TaKaRa). Expression levels were calculated using the 2^–ΔΔ*Ct*^ method. The following primers were used:

*IL-6* (forward: 5′-ACAGAAGGAGTGGCTAAGGA-3′; reverse: 5′-TTTCTGACCACAGTGAGGAA-3′);
*MCP-1* (forward: 5′-GCAAGATGATCCCAATGAGT-3′; reverse: 5′-TAGCTTCAGATTTACGGGTC-3′);
*MIP-2* (forward: 5′-AGTGAACTGCGCTGTCAATG-3′; reverse: 5′-CTTTGGTTCTTCCTTGAGG-3);
TNF-α (forward, 5′-CCCCAGTCTGTATCCTTCTAA-3′; reverse, 5′-TCGAGGCTCCAGTGAATT-3′);
β*-actin* (forward: 5′-GAGGTATCCTGACCCTGAAGTA-3′; reverse: 5′-CACACGCAGCTCATTGTAGA-3′).
Real-time qPCR results were normalized to β*-actin* expression.

### MILLIPLEX^®^ MAP and Enzyme-Linked Immunosorbent Assays

EMD Millipore’s MILLIPLEX^®^ MAP Mouse High Sensitivity *T* Cell Magnetic Bead Panel (Cell Signaling Technology) was used to simultaneously quantify any or all of the following mouse cytokines in cell culture media: transforming growth factor (TGF)-β1, IL-1β, IL-6, monocyte chemoattractant protein (MCP)-1, MIP-2, and TNF-α.

### Immunofluorescence Microscopy

Cells were fixed with 4% paraformaldehyde, permeabilized with phosphate-buffered saline (PBS) containing 0.1% Triton X-100, blocked in PBS containing 0.1% Tween supplemented with 5% goat serum, and stained with rat anti-mouse F4/80 (1:100, Abcam), rabbit anti-mouse pMLKL (1:100; Cell Signaling Technology, Beverly, MA, United States), rabbit anti-mouse collagen I (1:100, Abcam), and rabbit anti-mouse collagen VI (1:100, Abcam) primary antibodies. Coverslips were then sequentially labeled with species-specific fluorochrome-conjugated secondary antibodies and DAPI (Sigma-Aldrich). Samples were visualized using a fluorescence microscope (Olympus). Collagen I and collagen VI fluorescence per cell was analyzed using ImageJ software.

### Western Blot Analysis

Total cell lysates of cultured cells were prepared using M-PER Mammalian Protein Extraction Reagent (Thermo Fisher Scientific, Runcorn, Cheshire, United Kingdom). Primary antibodies against pRIPK3, RIPK3, pMLKL, and MLKL from a Mouse Reactive Necroptosis Antibody Sampler Kit (Cell Signaling Technology) were used. After incubation with secondary antibodies, immunocomplexes were detected using a WesternBreeze Chemiluminescent Detection Kit (WB7108; Thermo Fisher Scientific). β-actin was used as an internal control.

### Statistical Analyses

Data were analyzed using GraphPad Prism statistical software (GraphPad Software, Inc., La Jolla, CA, United States). Data are presented as mean ± standard deviation. An independent-sample *t* test or a one-way or two-way analysis of variance with Bonferroni’s *post hoc* analysis was performed as appropriate. *p*-values < 0.05 were considered statistically significant.

## Results

### The Volume of Fat Grafts Decreases Over Time in a Mouse Model

**FIGURE 1:**
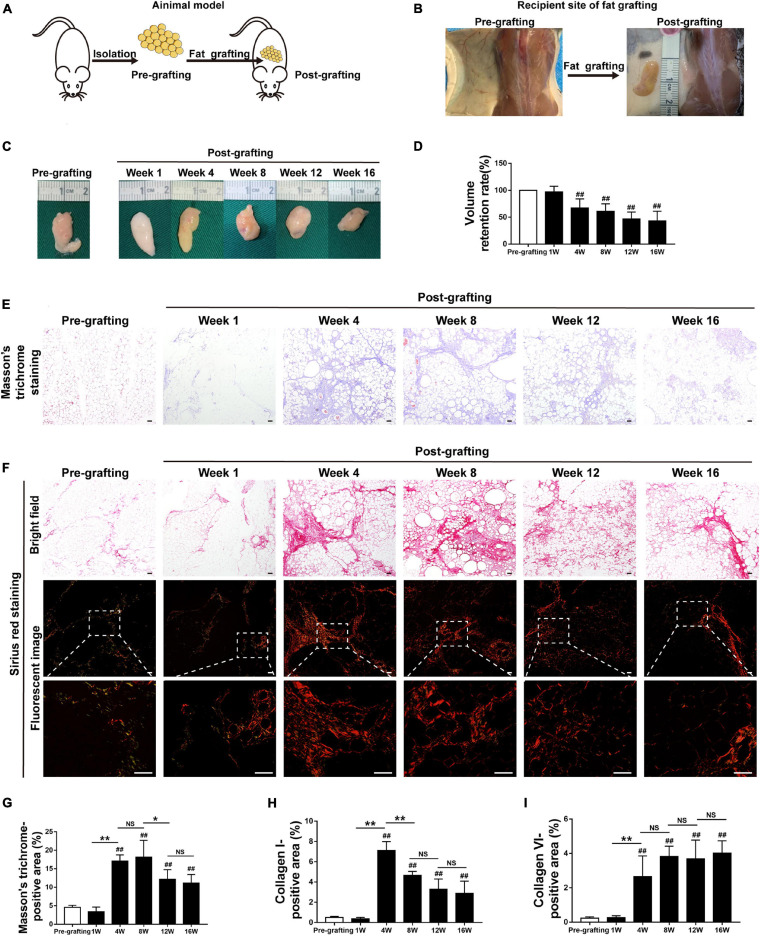
Adipose tissue fibrosis is increased in fat graft tissue. **(A,B)** Illustration of the animal model. **(C)** Macroscopic views of harvested fat tissue. **(D)** The retention volume of the fat grafts over time. **(E)** Representative Masson’s trichrome staining of fat tissue before and after grafting. **(F)** Representative Sirius red staining of fat tissue before and after grafting. **(G–I)** Quantification of the areas positive for Masson’s trichrome **(G)**, collagen I **(H)**, and collagen VI **(I)** staining in fat tissue before and after grafting. Scale bars = 50 μm. Data are presented as the mean ± SD. ^##^*P* < 0.01 compared with the pre-grafting group; *P < 0.05, **P < 0.01.

Fat tissue was harvested from the inguinal fat pads of mice and dissected into small pieces, which were grafted onto the backs of recipient mice (Figures 1A,B).

The implanted fat tissue was harvested, and graft volume was evaluated at different time points after fat grafting. Fat grafts showed a tougher texture than normal fat tissue (pre-grafting) and were covered with a thin, well-vascularized fibrous capsule from week 4 to week 16 (Figure 1C). The volume of fat grafts decreased in a time-dependent manner. The fat graft retention rate (final volume/initial volume) was approximately 42.89 ± 7.34% on week 16 (Figure 1D).

### Adipose Tissue Fibrosis Is Increased in Fat Graft Tissue

Changes to the ECM in fat tissue after fat grafting were examined by Masson’s trichrome staining. Interstitial fibrosis was extensive in fat tissue after grafting. It was increased at week 4–16 relative to pre-grafting, peaked at week 8, slightly decreased at week 12, and relatively stable at week 16 (Figures 1E,G). Collagen was detected by Sirius red staining. Collagen I fibers appeared red/yellow and collagen III fibers appeared green in Sirius red-stained paraffin sections underneath a polarized light microscope. Collagen I fibers were the main collagen fibers in grafts (Figure 1F). Immunohistochemical staining confirmed that the levels of collagen I and VI fibers were markedly higher after grafting than before grafting (Figures 1F,H,I and Supplementary Figure 1A). Consistently, expression of S100A4, which is mainly expressed by fibroblasts in fat tissue (Hou et al., 2018; Li et al., 2020), was significantly higher in fibrotic regions of fat grafts after grafting than before grafting (Supplementary Figure 1B). Double immunofluorescence staining of collagen I fibers (red) and S100A4 (green) demonstrated that fibroblasts and fibrotic regions were located adjacent to each other (Supplementary Figure 1C). Collectively, these observations indicate that fibrosis in fat tissue is higher after grafting than before grafting.

### MFCs Are Formed in CLSs in Fibrotic Regions of Fat Graft Tissue

**FIGURE 2:**
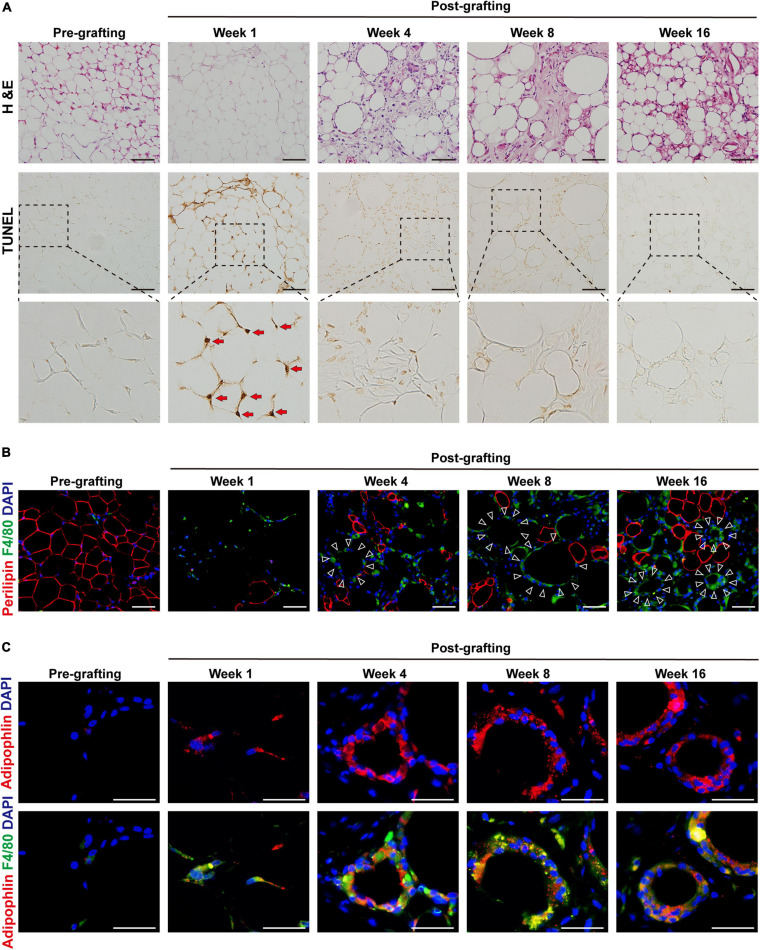
MFCs are formed in crown-like structures (CLSs) in fibrotic regions of fat graft tissue. **(A)** Representative H&E and TUNEL staining of fat tissue before and after grafting. Red arrows indicate the apoptotic adipocytes. **(B)** Representative immunofluorescence staining of perilipin (red) and F4/80 (green) in fat tissue before and after grafting. Large oil droplets or apoptotic adipocytes were surrounded by F4/80 + macrophages, forming typical CLSs (triangles). **(C)** Representative immunofluorescence staining of adipophilin (red) and F4/80 (green) in fat tissue before and after grafting. Scale bars = 50 μm.

Because aberrant inflammation is closely related to tissue fibrosis, we next explored the role of inflammation in fat graft fibrosis. Analysis of serial sections by H&E staining and the TUNEL apoptosis assay (Figure 2A) showed marked disruption of the adipose tissue architecture in fat grafts accompanied by a marked increase in adipocyte apoptosis at the early stage after fat grafting (weeks 1–4). Loss of adipocytes caused infiltration of macrophages into fat graft tissue, as determined by the upregulation of the macrophages markers F4/80 (Figure 2B). The number of macrophages infiltrating the grafts was appreciably higher than that of normal fat tissue (pre-grafting; Figure 2B). Macrophages clustered around apoptotic adipocytes or large oil droplets to form a typical CLS, in which macrophages scavenge the residual lipid droplets of apoptotic adipocytes (Figure 2B). Numerous macrophages in CLSs in fat graft tissue had an increased number of intracellular lipid droplets and were defined as MFCs. MFCs were subjected to immunofluorescence staining against adipophilin, a macrophage lipid droplet coat protein (Ouimet et al., 2011; Figure 2C).

### Necroptosis Is Activated in MFCs in Fat Graft Tissue

**FIGURE 3:**
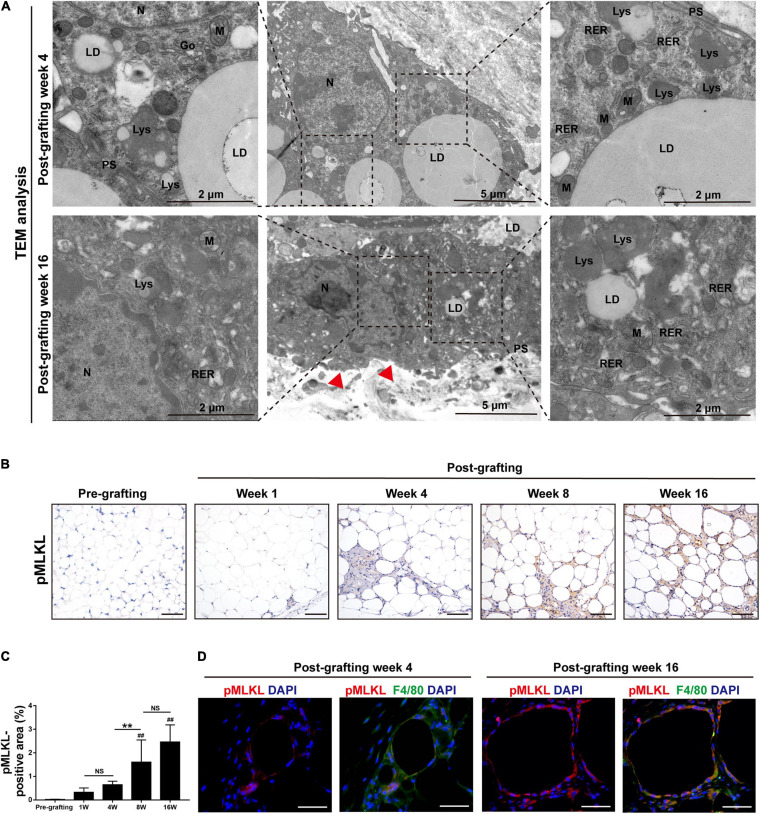
Necroptosis is activated in MFCs in fat graft tissue. **(A)** TEM of the ultrastructure of MFCs located in fat tissue after grafting. N, nucleus; LD, lipid droplet; PS, pseudopodia; M, mitochondria; RER, rough endoplasmic reticulum; and Lys, lysosome. Red arrowheads indicated the damaged plasma membrane. **(B,C)** Representative pMLKL staining **(B)** and quantification of the pMLKL-positive area **(C)** in fat tissue before and after grafting. **(D)** Representative immunofluorescence staining of pMLKL (red) and F4/80 (green) in fat tissue after grafting. Scale bars = 50 μm. Data are presented as the mean ± SD. ^##^*P* < 0.01 compared with the pre-grafting group; **P < 0.01.

The ultrastructure of macrophages in fat grafts was examined by TEM. Macrophages are characterized by high organelle complexity. They have a high content of mitochondria, lysosomes, Golgi bodies, and endoplasmic reticulum, as well as numerous pseudopodia. Macrophages in fat grafts on week 4 contained many intracellular lipid droplets and lipid droplet-ladened electron-dense vesicles resembling lysosomes. However, MFCs on week 16 exhibited the typical morphological features of necroptosis (Tian et al., 2016), including mitochondrial swelling and dissolution, cytoplasmic vacuolation, endoplasmic reticulum dilation, a severely damaged plasma membrane (Figure 3A).

The findings suggested that cellular lipids induce cell necrosis in MFCs. We therefore examined the activation of necroptosis in fat grafts. Expression of the necroptosis marker pMLKL was determined by immunohistochemical staining (Figure 3B). The area of pMLKL-positive cells in fibrotic depots of fat tissue was significantly larger at weeks 8 and 16 after grafting than before grafting (Figure 3C). Double immunofluorescence staining of pMLKL (red) and F4/80 (green) revealed that some macrophages underwent necroptosis after grafting (Figure 3D). These data indicate that activation of necroptosis in MFCs is involved in fat graft fibrosis.

### *In vitro* Apoptotic Adipocyte Model Recapitulates Adipocyte Apoptosis in Fat Grafts

**FIGURE 4:**
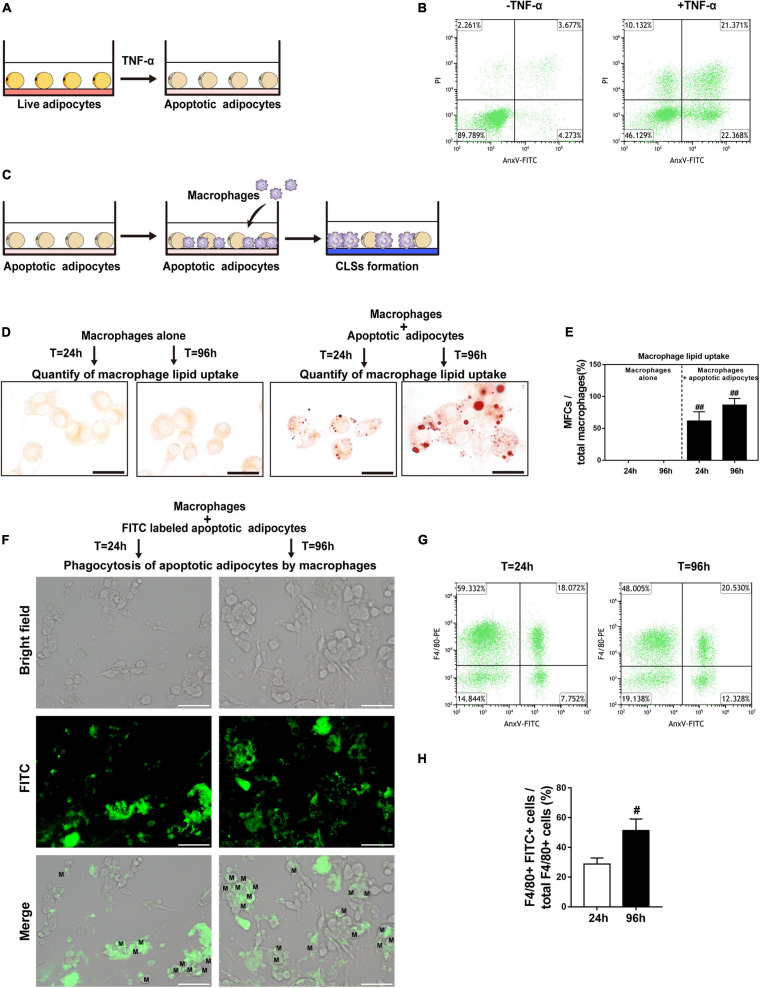
CLS cell culture model mirrors *in vivo* CLS morphology and shows formation of MFCs. **(A)** Illustration of induction of adipocyte apoptosis by TNF-α *in vitro*. **(B)** Double staining with AnxV-FITC and propidium iodide (PI) was used to determine the proportion of apoptotic (AnxV + /PI) adipocytes after apoptosis induction (right panel); uninduced adipocytes were used as controls (left panel). **(C)** Illustration of the CLS cell culture model *in vitro*: RAW 264.7 macrophages were incubated with TNF-α-induced apoptotic adipocytes to form CLSs, and RAW 264.7 macrophages incubated alone were used as controls. **(D,E)** MFCs are formed in the CLS cell culture model *in vitro.* **(D)** Representative Oil Red O staining. **(E)** Macrophage lipid uptake was quantified as the percentage of MFCs at 24 and 96 h. **(F–H)** The phagocytosis of apoptotic adipocytes by RAW 264.7 macrophages was detected by immunofluorescence staining and flow cytometry. **(F)** RAW 264.7 macrophages were incubated with apoptotic adipocytes stained by FITC-conjugated AnxV for 24 h or 96 h. FITC + macrophages indicate the phagocytosis of apoptotic adipocytes by those macrophages. **(G,H)** The macrophage phagocytic rate at 24 h or 96 h was quantitatively analyzed by flow cytometry; phagocytic macrophages were double-positive for PE (labeled F4/80) and FITC (labeled apoptotic adipocytes). Scale bars = 15 μm in **(D)** and Scale bars = 50 μm in **(F)**. M, macrophage. Data are presented as the mean ± SD. ^#^*P* < 0.05, ^ ##^*P* < 0.01 compared with the CLS cell culture model group at 24 h.

The differentiated 3T3-L1 adipocytes were cultured in medium supplemented with TNF-α to induce apoptosis (Figure 4A). TNF-α is a pro-inflammatory cytokine that is increased in fat grafts and induces adipocyte apoptosis (Mok et al., 2018). The apoptotic adipocytes were quantitatively analyzed by flow cytometry after double staining with PI and FITC-conjugated AnxV (Figure 4B). After incubation for 24 h, >40% of the cells underwent apoptosis, whereas 10% of the cells underwent necrosis (Figure 4B). To determine whether necroptosis occurred under these conditions, adipocytes were co-cultured with TNF-α in the presence or absence of a necroptosis inhibitor (Nec-1 or GSK872) for 24 h (Supplementary Figure 2A). PI staining was used to detect adipocyte necrosis, which showed that treatment with a necroptosis inhibitor did not decrease the number of PI-positive cell in the *in vitro* apoptotic adipocyte model (Supplementary Figure 2B). These data indicate that TNF-α was sufficient to induce apoptosis but not necroptosis in adipocytes *in vitro.*

### Lipid Accumulation in CLS Macrophages Causes Formation of MFCs *in vitro*

To further investigate the interactions of macrophages with apoptotic adipocytes, we used a physiologically relevant co-culture assay (Figure 4C) that mimics formation of CLSs in fat grafts. In this model, apoptotic adipocytes were incubated with RAW 264.7 macrophages to induce the formation of CLSs. Macrophages were cultured alone as controls. Oil Red O staining was used to detect lipid uptake in macrophages (Figure 4D). Approximately 86% of CLS macrophages contained lipid droplets after 96 h of co-culture, indicating that these macrophages were converted into MFCs after 96 h of co-culture with apoptotic adipocytes (Figure 4E).

To perform phagocytosis assays, FITC-conjugated AnxV-labeled apoptotic adipocytes were co-cultured with RAW 264.7 macrophages for 24 h or 96 h. Fragments of apoptotic adipocytes were observed in macrophages by fluorescence microscopy, demonstrating that macrophages specifically phagocytosed apoptotic adipocytes (Figure 4F). In addition, FITC-conjugated AnxV-labeled apoptotic adipocytes and macrophages tagged with PE-conjugated antibodies against F4/80 were co-cultured to confirm the engulfment of apoptotic adipocytes by macrophages by flow cytometry (Figures 4G,H). FITC + F4/80 + cells were considered as macrophage cells that had phagocytosed FITC-conjugated AnxV-labeled apoptotic adipocytes. The percentage of FITC + F4/80 + cells increased from 24 to 96 h of incubation. These data indicate that macrophages effectively phagocytosed apoptotic adipocytes and formed MFCs in the CLS cell culture model.

### Necroptosis Is Activated in MFCs in the CLS Cell Culture Model

**FIGURE 5:**
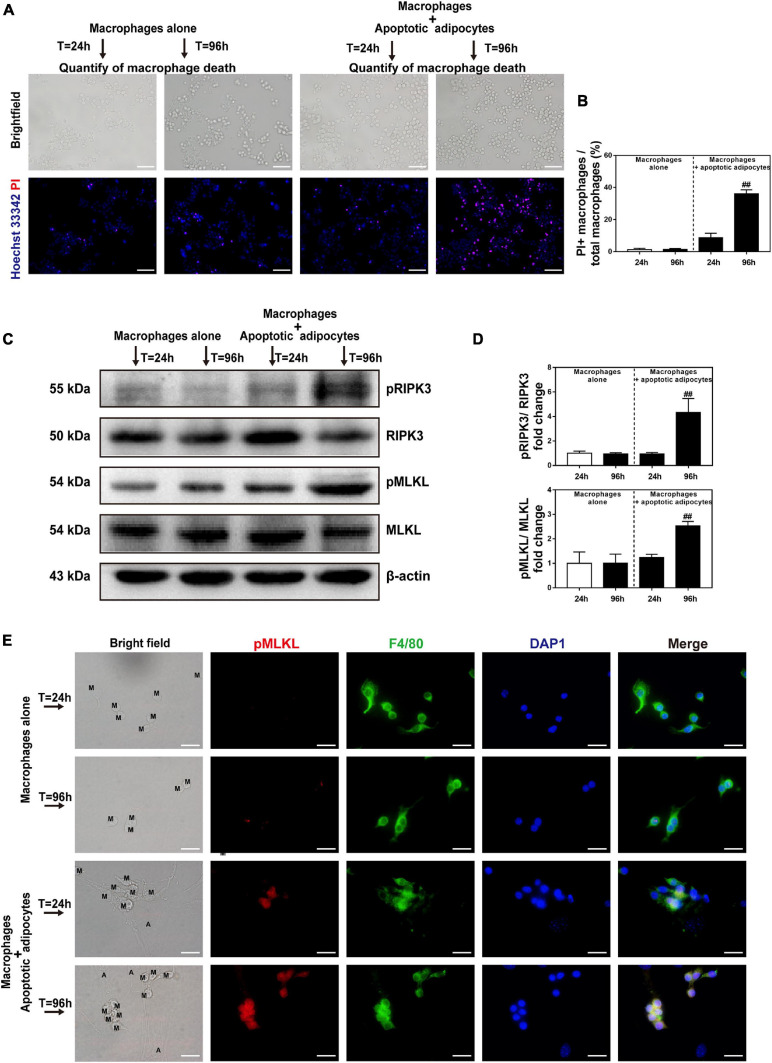
Necroptosis is activated in MFCs in the *in vitro* CLS cell culture model. **(A,B)** Necrosis of macrophages determined by Hoechst 33342 and PI staining in the CLS cell culture group and in RAW 264.7 macrophages cultured alone at 24 and 96 h. **(A)** Hoechst 33342 + PI + macrophages in the pictures indicate necrotic macrophages. **(B)** Quantification of macrophage necrosis in both groups at 24 and 96 h. **(C,D)** Western blot analyses of MLKL, pMLKL, RIPK3, and pRIPK3 in the CLS cell culture group and in RAW 264.7 macrophages cultured alone at 24 and 96 h. **(E)** Immunofluorescence staining of pMLKL (red) and F4/80 (green) in the CLS cell culture group and in RAW 264.7 macrophages cultured alone at 24 and 96 h. Scale bars = 50 μm. A, adipocyte; M, macrophage. Data are presented as the mean ± SD. ^##^*P* < 0.01 compared with the group of RAW 264.7 macrophages cultured alone at 24 h.

To explore the pathological process of CLS macrophages *in vitro*, Hoechst 33342/PI staining was used to determine whether cell necrosis occurred in CLS macrophages. CLS macrophages can be distinguished from adipocytes under bright field observation. They are smaller than adipocytes and appear as small, round, or short spindle-shaped cells, whereas adipocytes are large, round, full-lipid cells. Co-culture with apoptotic adipocytes time-dependently increased the number of PI and Hoechst 33342 positive necrotic CLS macrophages, and approximately 36% of CLS macrophages underwent necrosis in co-culture at 96 h (Figures 5A,B). The results of western blotting demonstrated that expression of the necroptosis-related proteins pRIPK3 and pMLKL markedly increased from 24 to 96 h in the CLS cell culture model (Figures 5C,D). In addition, immunofluorescence staining for F4/80 and pMLKL showed that pMLKL was expressed specifically in CLS macrophages but not in CLS adipocytes (Figure 5E). These results indicate that necroptosis of CLS macrophages was induced after 96 h of incubation.

**FIGURE 6:**
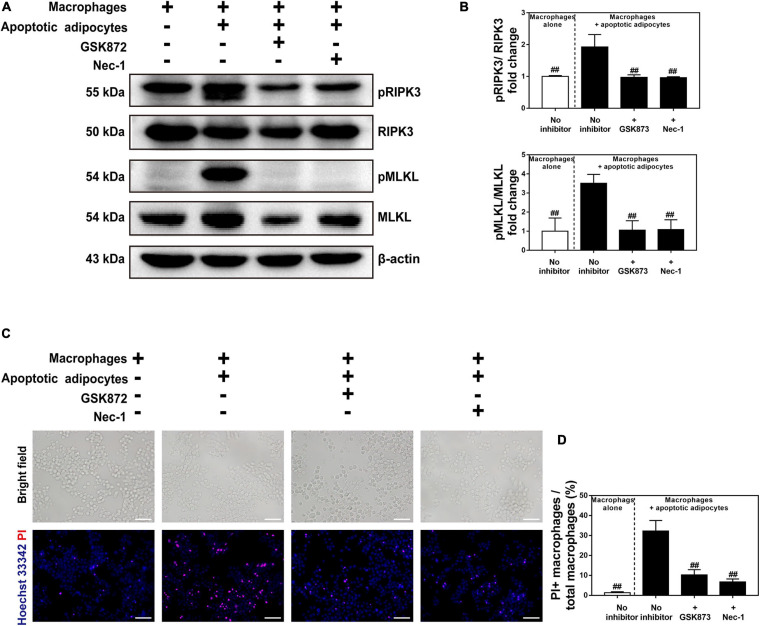
Necroptosis inhibitors suppressed necroptosis of MFCs in the *in vitro* CLS cell culture model. **(A,B)** Western blot analyses of MLKL, pMLKL, RIPK3, and pRIPK3 in four groups, including RAW 264.7 macrophages cultured alone, and those in CLS cell culture incubated in the presence or absence of a necroptosis inhibitor (Nec-1 or GSK872) at 96 h. **(C,D)** Necrosis of macrophages determined by Hoechst 33342 and PI staining in four groups of RAW 264.7 macrophages cultured alone, and those in CLS cell culture incubated in the presence or absence of a necroptosis inhibitor (Nec-1 or GSK872) at 96 h. Scale bars = 50 μm. Data are presented as the mean ± SD. ^##^*P* < 0.01 compared with the CLS cell culture model group incubated without necroptosis inhibitors.

To confirm the presence of CLS macrophages, macrophages were co-cultured with apoptotic adipocytes in the presence or absence of a necroptosis inhibitor (Nec-1 or GSK872) for 96 h. Macrophages were cultured alone as a control group. Western blot detection of pRIPK3 and pMLKL confirmed that GSK872 and Nec-1 markedly inhibited the necroptotic pathway in CLS macrophages (Figures 6A,B). Treatment with GSK872 or Nec-1 reduced the number of PI and Hoechst 33342 positive necrotic CLS macrophages (Figures 6C,D). Because 86% of CLS macrophages formed MFCs, and approximately 64% of CLS macrophages survived in the CLS cell culture model, it can be concluded that necroptosis occurred in MFCs in the CLS cell culture model.

### Necroptosis of MFCs Induces Collagen Expression in Fibroblasts via a Paracrine Mechanism

**FIGURE 7:**
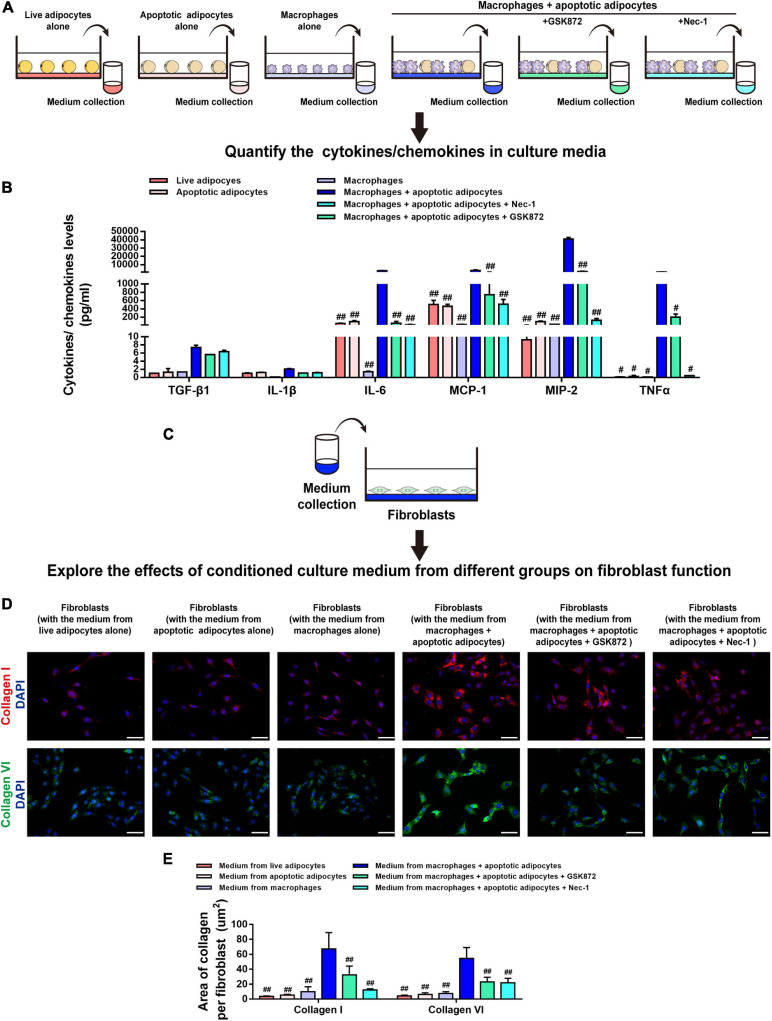
Necroptosis of MFCs induces collagen expression in fibroblasts via a paracrine mechanism. **(A,B)** MILLIPLEX MAP assays were used to detect the expression levels of cytokines/chemokines in conditioned media collected from six groups, including adipocytes (live or apoptotic), RAW 264.7 macrophages, and CLS cultured cells incubated in the presence or absence of a necroptosis inhibitor (Nec-1 or GSK872). **(C)** Fibroblasts were treated with conditioned media collected from the six groups. **(D)** Immunofluorescence staining of collagen I and collagen VI in fibroblasts to investigate the paracrine effect of the culture media from various groups on fibroblasts. **(E)** Quantification of the collagen I- and collagen VI-positive areas per fibroblast. Scale bars = 50 μm. Data are presented as the mean ± SD. ^#^*P* < 0.05, ^##^*P* < 0.01 compared with the CLS cell culture model group incubated without necroptosis inhibitors.

Necroptosis strongly induces cytokine expression (Zhu et al., 2018), which led us to hypothesize that necroptosis of MFCs in the CLS cell culture model induces inflammation. To test this hypothesis, cell media from live or apoptotic 3T3-L1 adipocytes, macrophages cultured alone, and macrophages co-cultured with apoptotic adipocytes and treated with or without a necroptosis inhibitor (Nec-1 or GSK872) were collected, and the levels of cytokines/chemokines were analyzed using MILLIPLEX^®^ MAP assays (Figure 7A). As shown in Figure 7B, the expression of IL-6, TNF-α, MCP-1, and MIP-2 was lower in co-cultures treated with Nec-1 or GSK872 than in the untreated co-culture group (Figure 7B). Furthermore, cytokines/chemokines were seldom released in the control groups, including live or apoptotic 3T3-L1 adipocytes and macrophages cultured alone (Figure 7B). These data demonstrate that necroptosis of MFCs in the CLS cell culture model induced the production of proinflammatory cytokines/chemokines.

Next, fibroblasts were treated with conditioned media from the six groups, and collagen expression was analyzed by staining for collagen I and collagen VI (Figure 7C). Immunofluorescence staining demonstrated that, in contrast to the culture medium from live 3T3-L1 adipocytes, apoptotic 3T3-L1 adipocytes, or macrophages, the levels of collagen I and collagen VI fibers expressed by fibroblasts were markedly increased by treatment with medium from the CLS cell culture model and decreased by addition of GSK872 or Nec-1 (Figures 7D,E).

### Blockade of Necroptosis Alleviates Fat Graft Fibrosis *in vivo*

**FIGURE 8:**
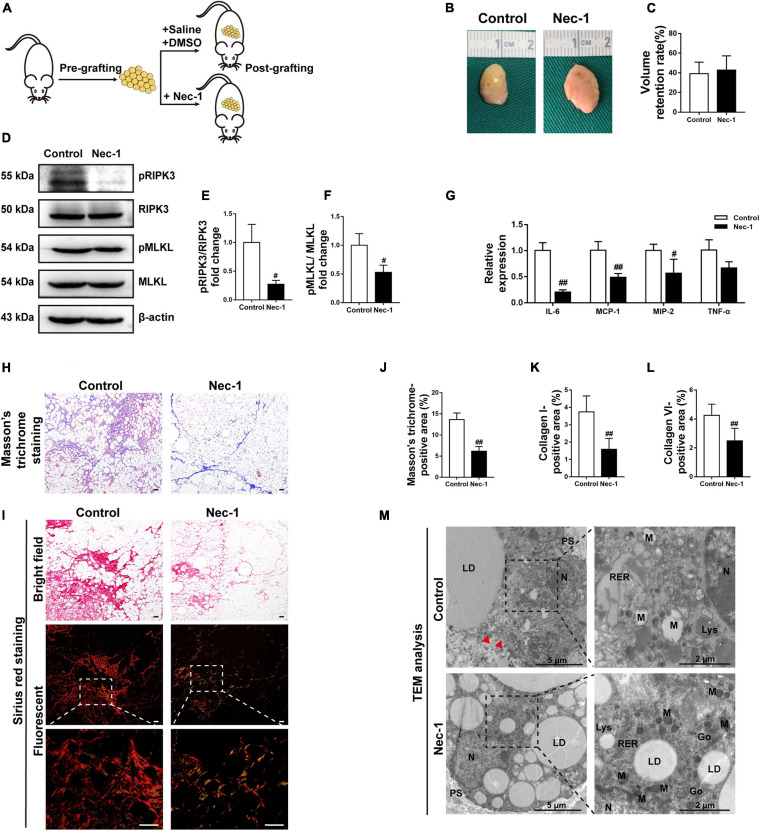
Blockade of necroptosis alleviates fat graft fibrosis. **(A)** Illustration of the animal model. Fat grafting model mice were administered Nec-1 or vehicle. **(B)** Macroscopic views of harvested fat tissue. **(C)** The retention volume of the fat grafts over time. **(D–F)** Western blot analyses of MLKL, pMLKL, RIPK3, and pRIPK3 *in vivo*. **(G)** The gene expression level of the cytokines TNF-α and IL-6 and the chemokines MCP-1 and MIP-2 in fat grafts. **(H)** Representative Masson’s trichrome staining of fat tissue at week 16 after grafting in the different groups. **(I)** Representative Sirius red staining of fat tissue at week 16 after grafting in the different groups. **(J–L)** Quantification of the areas positive for Masson’s trichrome **(J)**, collagen I **(K)**, and collagen VI **(L)** staining in fat tissue at week 16 after grafting in the different groups. Scale bars = 50 μm. Data are presented as the mean ± SD. ^#^*P* < 0.05, ^##^*P* < 0.01 compared with the control group. **(M)** TEM analyses of the ultrastructure of MFCs in fat tissue at week 16 after grafting in the different groups. N, nucleus; LD, lipid droplet; PS, pseudopodia; M, mitochondria; RER, rough endoplasmic reticulum; Go, golgiosome; Lys, lysosome; and Red arrowheads indicated the damaged plasma membrane.

Because Nec-1 had a stronger effect on reducing collagen expression in fibroblasts *in vitro* than GSK872, fat grafting model mice were treated with Nec-1 or vehicle to explore the biological effects of necroptosis on the development of fat graft fibrosis *in vivo* (Figure 8A). The implanted fat tissue was harvested (Figure 8B), and the graft volume was evaluated on week 16 in both groups (Figure 8C). The volume retention rate did not differ significantly between the two groups. However, western blot analysis of pRIPK3 and pMLKL confirmed that Nec-1 markedly inhibited the necroptotic pathway in fat grafts on week 16 (Figures 8D–F). Furthermore, Nec-1 significantly downregulated the IL-6 cytokine and the chemokines, including MCP-1 and MIP-2, in fat grafts (Figure 8G).

Nec-1 treatment significantly reduced the areas positively stained with Masson’s trichrome and Sirius red at 16 weeks after grafting (Figures 8H,J). Consistently, the areas positive for collagen I and collagen VI were significantly smaller in Nec-1-treated mice than in vehicle-treated mice (Figures 8I,K,L and Supplementary Figure 3). Ultrastructural analysis of fat grafts using TEM in vehicle-treated mice showed typical necrotic changes, which were markedly ameliorated in Nec-1-treated mice (Figure 8M).

## Discussion

**FIGURE 9:**
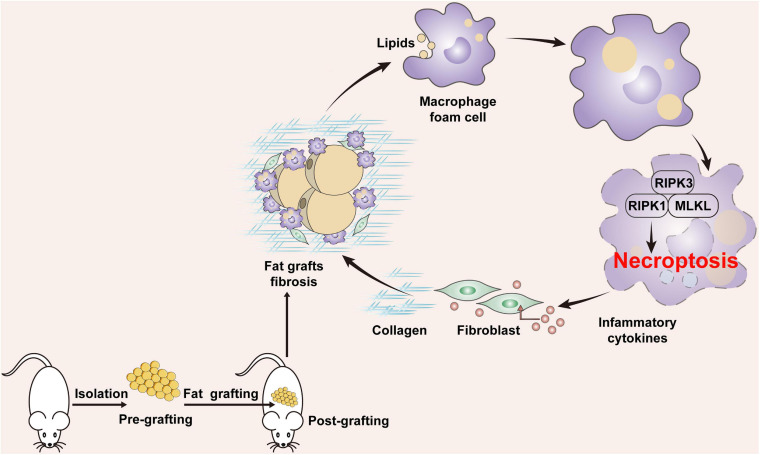
Potential role of MFC necroptosis in fat graft fibrosis. After fat grafting, lipids released from apoptotic adipocytes induce formation of MFCs and an increased level of cellular lipids mediates necroptosis of MFCs, which upregulates the expression of proinflammatory cytokines/chemokines, leading to collagen synthesis by fibroblasts. Consequently, overproduction of collagens leads to fat tissue fibrosis after grafting.

Fibrosis is a major complication of fat grafting and is implicated in the declined expandability and impaired function of adipose tissue. However, little is known about how adipose tissue fibrosis develops after grafting. This study provides evidence that necroptosis of MFCs leads to progression of fat graft fibrosis. After grafting, lipids released from apoptotic adipocytes induce formation of MFCs, and an increased level of lipids mediates necroptosis of MFCs, which upregulates the expression of proinflammatory cytokines/chemokines, leading to collagen synthesis by fibroblasts. Consequently, overproduction of collagens leads to fat tissue fibrosis after grafting. Blockade of necroptosis alleviated fat graft fibrosis (Figure 9).

Fibrogenesis is a complex process caused by a variety of cells including fibroblasts, immune cells, and parenchymal cells. Fibroblasts are the main producers of ECM in other fibrosis-prone tissues (Datta et al., 2018); however, little is known about their role in adipose tissue fibrosis. In this study, the number of S100A4-positive cells (fibroblasts) was increased in fat grafts. Approximately 85% of stromal vascular fraction CD45-negative cells are anti-fibroblast (FIB)-positive cells in adipose tissue (Navarro et al., 2015), which are considered to be involved in ECM remodeling (Tanaka et al., 2014). Collagen I and collagen VI were highly expressed and their expression remained high for up to 16 weeks in fat grafts relative to before grafting. In particular, collagen I molecules were staggered and interwoven with each other to form thick collagen bundles around oil cysts or dead adipocytes in fat grafts, which is reportedly important for limitation of adipose tissue expansion (Guzmán-Ruiz et al., 2020). Similarly, increased collagen VI expression is implicated in obesity with poor metabolic outcomes (Sun et al., 2014; Hasegawa et al., 2018).

Adipose fibrosis and adipose ECM remodeling are associated with increased macrophage infiltration and upregulation of inflammation in adipose tissue (Spencer et al., 2010). In this context, we found that macrophage infiltration into fat grafts was closely related to graft fibrosis. These macrophages clustered around apoptotic adipocytes or large oil droplets to form typical CLSs. Notably, most macrophages in fibrotic depots were filled with cytosolic LDs, which resulted in their foamy appearance under a microscope, and were defined as MFCs. Moreover, the plasma membrane of MFCs was severely damaged, demonstrating the cell death of MFCs *in vivo*. This conclusion was further supported by immunohistochemical staining for pMLKL, a definitive biomarker of necroptosis activity *in vivo* (Jouan-Lanhouet et al., 2014; Karunakaran et al., 2016; Tonnus et al., 2019), and double immunofluorescence staining for pMLKL and F4/80. We confirmed that necroptosis, an emerging pathway underlying inflammatory cell death, was active within MFCs in fibrotic regions of fat grafts at the late stage after grafting.

Necroptosis has been implicated in various pathophysiological conditions, including myocardial (Karunakaran et al., 2016; Chen et al., 2020), liver (Choi et al., 2015), pulmonary (Sauler et al., 2019), and renal (Shi et al., 2020) fibrosis. Expression of pRIPK3 and pMLKL is tightly correlated with the degree of necroptosis (Dondelinger et al., 2014), and induction of pRIPK3 expression is sufficient to trigger necroptotic cell death (Zhang et al., 2009). Here, we found for the first time that MFCs underwent necroptosis in fibrotic regions of grafts in a time-dependent manner and that expression of pMLKL was significantly increased in MFCs. We next developed a physiologically relevant *in vitro* assay in which macrophages were co-cultured with apoptotic 3T3-L1 adipocytes, leading to formation of MFCs with accumulation of adipocyte-derived lipids. We found that apoptotic adipocytes were sufficient to induce necroptotic death of MFCs, as evidenced by activation of the RIPK3/MLKL signaling pathway and attenuation of this cell death upon treatment with specific necroptosis inhibitors.

Based on our observations, we hypothesized that necroptosis of MFCs play a role in graft inflammation and affect fibrogenesis. Necroptosis is an important trigger of inflammation. In sterile settings, necroptotic cells can directly trigger inflammation by releasing proinflammatory cytokines (Pasparakis and Vandenabeele, 2015). In addition, RIPK3/MLKL-dependent necroptosis can trigger activation of the inflammasome by inducing changes in the redox state, intracellular ion concentrations, and metabolic status of the cell, all of which are well-known inflammasome inducers (Lamkanfi and Dixit, 2014). Consistently, our data suggest that MFCs undergo necroptotic death in the presence of dead adipocytes in a co-culture assay, release inflammatory cytokines and chemokines including TNF-β, TNF-α, IL-1α, IL-6, and MCP-1 into the extracellular space, and thereby increase expression of fibrosis-related genes and collagens in fibroblasts via a paracrine mechanism. Cytokines such as those mentioned above cause damage of metabolic organs and drive tissue fibrosis (Turner et al., 2007; Gharaee-Kermani et al., 2012). Necroptotic signaling can promote the secretion of proinflammatory cytokines in a cell-intrinsic manner, especially through the inflammasome pathway (Conos et al., 2017). The functional output of inflammasome activation includes increased secretion of IL-1β (Martinon et al., 2002; Lawlor et al., 2015; Sharma and Kanneganti, 2016). In this study, IL-1β was expressed at extremely low levels and its expression did not change significantly. Therefore, the present results indicate that RIPK3/MLKL-dependent necroptosis, rather than inflammasome activation, may lead to the secretion of inflammatory cytokines and chemokines in MFCs.

Taken together, it is plausible that reducing necroptosis of MFCs will decrease inflammation and fibrogenesis in fat grafts. Therefore, we next sought to attenuate fat graft fibrosis *in vivo* by reducing necroptotic death of MFCs and demonstrated that Nec-1 treatment reduced fat graft fibrosis. Studies of necroptosis have shown that intraperitoneal injection of Nec-1 protects cellular structures under necroptosis-related pathological conditions in animal models (Liang et al., 2019). This is the first report of the long-term therapeutic application of Nec-1 to reduce fat graft fibrosis. Our data highlight the paracrine loop between MFCs and fibroblasts, which establishes a vicious process that augments inflammatory changes and fibrogenesis in adipose tissue after grafting.

## Conclusion

In summary, this study demonstrated that necroptosis of MFCs induced by apoptotic adipocytes plays a critical role in fat graft inflammation and may promote the progression of graft fibrosis by inducing collagen secretion in fibroblasts. Thus, blockade of necroptosis in macrophages is a potential approach to prevent fibrosis in fat grafts.

## Data Availability Statement

The original contributions presented in the study are included in the article/Supplementary Material, further inquiries can be directed to the corresponding authors.

## Ethics Statement

The animal study was reviewed and approved by Animal Care and Use Committee of Nanfang Hospital.

## Author Contributions

YY and FL: conception and design, financial support, and final approval of the manuscript. XC and ZD: conception and design, manuscript writing, assembly of data, and data analysis and interpretation. JF: assembly of data. QC: data analysis and interpretation. All authors contributed to the article and approved the submitted version.

## Conflict of Interest

The authors declare that the research was conducted in the absence of any commercial or financial relationships that could be construed as a potential conflict of interest.

## References

[B1] Boada-Romero E. Martinez J. Heckmann B. L. Green D. R. (2020). The clearance of dead cells by efferocytosis. *Nat Rev Mol Cell Biol* 21 398–414. 10.1038/s41580-020-0232-1 32251387 PMC7392086

[B2] Caccamo A. Branca C. Piras I. S. Ferreira E. Huentelman M. J. Liang W. S. (2017). Necroptosis activation in Alzheimer’s disease. *Nat Neurosci* 20 1236–1246. 10.1038/nn.4608 28758999

[B3] Cai J. Li B. Liu K. Li G. Lu F. (2017). Macrophage infiltration regulates the adipose ECM reconstruction and the fibrosis process after fat grafting. *Biochem Biophys Res Commun* 490 560–566. 10.1016/j.bbrc.2017.06.078 28625922

[B4] Chen H. Tang L. J. Tu H. Zhou Y. J. Peng J. (2020). Arctiin protects rat heart against ischemia/reperfusion injury via a mechanism involving reduction of necroptosis. *Eur J Pharmacol* 875 173053. 10.1016/j.ejphar.2020.173053 32135123

[B5] Choi H. S. Kang J. W. Lee S. M. (2015). Melatonin attenuates carbon tetrachloride-induced liver fibrosis via inhibition of necroptosis. *Transl Res* 166 292–303. 10.1016/j.trsl.2015.04.002 25936762

[B6] Coats B. R. Schoenfelt K. Q. Barbosa-Lorenzi V. C. Peris E. Cui C. Hoffman A. (2017). Metabolically Activated Adipose Tissue Macrophages Perform Detrimental and Beneficial Functions during Diet-Induced Obesity. *Cell Rep* 20 3149–3161. 10.1016/j.celrep.2017.08.096 28954231 PMC5646237

[B7] Condé-Green A. Marano A. A. Lee E. S. Reisler T. Granick M. S. (2015). Fat Grafting and Adipose-Derived Regenerative Cells in Burn Wound Healing and Scarring: A Systematic Review of the Literature. *Plast Reconstr Surg* 137 302–312. 10.1097/PRS.0000000000001918 26710034

[B8] Conos S. A. Chen K. W. De Nardo D. Hara H. Whitehead L. Núñez G. (2017). Active MLKL triggers the NLRP3 inflammasome in a cell-intrinsic manner. *Proc Natl Acad Sci U S A* 114 E961–E969. 10.1073/pnas.1613305114 28096356 PMC5307433

[B9] Datta R. Podolsky M. J. Atabai K. (2018). Fat fibrosis: friend or foe? *JCI Insight* 3 e122289. 10.1172/jci.insight.122289 30282827 PMC6237440

[B10] Dondelinger Y. Declercq W. Montessuit S. Roelandt R. Goncalves A. Bruggeman I. (2014). MLKL compromises plasma membrane integrity by binding to phosphatidylinositol phosphates. *Cell Rep* 7 971–981. 10.1016/j.celrep.2014.04.026 24813885

[B11] Eto H. Kato H. Suga H. Aoi N. Doi K. Kuno S. (2012). The fate of adipocytes after nonvascularized fat grafting: evidence of early death and replacement of adipocytes. *Plast Reconstr Surg* 129 1081–1092. 10.1097/PRS.0b013e31824a2b19 22261562

[B12] Evans T. D. Jeong S. J. Zhang X. Sergin I. Razani B. (2018). TFEB and trehalose drive the macrophage autophagy-lysosome system to protect against atherosclerosis. *Autophagy* 14 724–726. 10.1080/15548627.2018.1434373 29394113 PMC5959328

[B13] Gharaee-Kermani M. Kasina S. Moore B. B. Thomas D. Mehra R. Macoska J. A. (2012). CXC-type chemokines promote myofibroblast phenoconversion and prostatic fibrosis. *PLoS One* 7:e49278. 10.1371/journal.pone.0049278 23173053 PMC3500280

[B14] Guglielmi V. Cardellini M. Cinti F. Corgosinho F. Cardolini I. D’Adamo M. (2015). Omental adipose tissue fibrosis and insulin resistance in severe obesity. *Nutr Diabetes* 5 e175. 10.1038/nutd.2015.22 26258766 PMC4558556

[B15] Guzmán-Ruiz R. Tercero-Alcázar C. Rabanal-Ruiz Y. Díaz-Ruiz A. El Bekay R. Rangel-Zuñiga O. A. (2020). Adipose tissue depot-specific intracellular and extracellular cues contributing to insulin resistance in obese individuals. *Faseb j* 34 7520–7539. 10.1096/fj.201902703R 32293066 PMC7384030

[B16] Hagit Shapiro Tal Pecht Ruthy Shaco-Levy (2013). Adipose tissue foam cells are present in human obesity. *J Clin Endocrinol Metab* 98 1173–1181. 10.1210/jc.2012-2745 23372170

[B17] Haka A. S. Barbosa-Lorenzi V. C. Lee H. J. Falcone D. J. Hudis C. A. Dannenberg A. J. (2016). Exocytosis of Macrophage Lysosomes Leads to Digestion of Apoptotic Adipocytes and Foam Cell Formation. *Journal of Lipid Research* 57 980–992. 10.1194/jlr.m064089 27044658 PMC4878183

[B18] Hasegawa Y. Ikeda K. Chen Y. Alba D. L. Stifler D. Shinoda K. (2018). Repression of Adipose Tissue Fibrosis through a PRDM16-GTF2IRD1 Complex Improves Systemic Glucose Homeostasis. *Cell Metab* 27 180.e–194.e. 10.1016/j.cmet.2017.12.005 29320702 PMC5765755

[B19] Hou S. Jiao Y. Yuan Q. Zhai J. Tian T. Sun K. (2018). S100A4 protects mice from high-fat diet-induced obesity and inflammation. *Lab Invest* 98 1025–1038. 10.1038/s41374-018-0067-y 29789685

[B20] Jouan-Lanhouet S. Riquet F. Duprez L. Vanden Berghe T. Takahashi N. Vandenabeele P. (2014). Necroptosis, in vivo detection in experimental disease models. *Semin Cell Dev Biol* 35 2–13. 10.1016/j.semcdb.2014.08.010 25160988

[B21] Kaczmarek A. Vandenabeele P. Krysko D. V. (2013). Necroptosis: the release of damage-associated molecular patterns and its physiological relevance. *Immunity* 38 209–223. 10.1016/j.immuni.2013.02.003 23438821

[B22] Karunakaran D. Geoffrion M. Wei L. Gan W. Richards L. Shangari P. (2016). Targeting macrophage necroptosis for therapeutic and diagnostic interventions in atherosclerosis. *Sci Adv* 2 e1600224. 10.1126/sciadv.1600224 27532042 PMC4985228

[B23] Katzel E. B. Bucky L. P. (2017). Fat Grafting to the Breast: Clinical Applications and Outcomes for Reconstructive Surgery. *Plast Reconstr Surg* 140 69s–76s. 10.1097/prs.0000000000003945 29064924

[B24] Klinger M. Marazzi M. Vigo D. Torre M. (2008). Fat injection for cases of severe burn outcomes: a new perspective of scar remodeling and reduction. *Aesthetic Plast Surg* 32 465–469. 10.1007/s00266-008-9122-1 18305985

[B25] Lamkanfi M. Dixit V. M. (2014). Mechanisms and functions of inflammasomes. *Cell* 157 1013–1022. 10.1016/j.cell.2014.04.007 24855941

[B26] Lawlor K. E. Khan N. Mildenhall A. Gerlic M. Croker B. A. D’Cruz A. A. (2015). RIPK3 promotes cell death and NLRP3 inflammasome activation in the absence of MLKL. *Nat Commun* 6 6282. 10.1038/ncomms7282 25693118 PMC4346630

[B27] Li Z. Li Y. Liu S. Qin Z. (2020). Extracellular S100A4 as a key player in fibrotic diseases. *J Cell Mol Med* 24 5973–5983. 10.1111/jcmm.15259 32307910 PMC7294136

[B28] Liang Y. X. Wang N. N. Zhang Z. Y. Juan Z. D. Zhang C. (2019). Necrostatin-1 Ameliorates Peripheral Nerve Injury-Induced Neuropathic Pain by Inhibiting the RIP1/RIP3 Pathway. *Front Cell Neurosci* 13:211. 10.3389/fncel.2019.00211 31156396 PMC6529821

[B29] Linkermann A. Heller J. O. Prókai A. Weinberg J. M. De Zen F. Himmerkus N. (2013). The RIP1-kinase inhibitor necrostatin-1 prevents osmotic nephrosis and contrast-induced AKI in mice. *J Am Soc Nephrol* 24 1545–1557. 10.1681/asn.2012121169 23833261 PMC3785275

[B30] Marcelin G. Ferreira A. Liu Y. Atlan M. Aron-Wisnewsky J. Pelloux V. (2017). A PDGFRα-Mediated Switch toward CD9(high) Adipocyte Progenitors Controls Obesity-Induced Adipose Tissue Fibrosis. *Cell Metab* 25 673–685. 10.1016/j.cmet.2017.01.010 28215843

[B31] Martinon F. Burns K. Tschopp J. (2002). The inflammasome: a molecular platform triggering activation of inflammatory caspases and processing of proIL-beta. *Mol Cell* 10 417–426. 10.1016/s1097-2765(02)00599-3 12191486

[B32] Mashiko T. Yoshimura K. (2015). How does fat survive and remodel after grafting? *Clin Plast Surg* 42 181–190. 10.1016/j..2014.12.008 25827562

[B33] Mineda K. Kuno S. Kato H. Kinoshita K. Doi K. Hashimoto I. (2014). Chronic inflammation and progressive calcification as a result of fat necrosis: the worst outcome in fat grafting. *Plast Reconstr Surg* 133 1064–1072. 10.1097/prs.0000000000000097 24776542

[B34] Mok H. Feng J. Hu W. Wang J. Cai J. Lu F. (2018). Decreased serum estrogen improves fat graft retention by enhancing early macrophage infiltration and inducing adipocyte hypertrophy. *Biochem Biophys Res Commun* 501 266–272. 10.1016/j.bbrc.2018.04.232 29729271

[B35] Navarro A. Marín S. Riol N. Carbonell-Uberos F. Miñana M. D. (2015). Fibroblast-Negative CD34-Negative Cells from Human Adipose Tissue Contain Mesodermal Precursors for Endothelial and Mesenchymal Cells. *Stem Cells Dev* 24 2280–2296. 10.1089/scd.2015.0013 26068131

[B36] Ouimet M. Franklin V. Mak E. Liao X. (2011). Autophagy Regulates Cholesterol Efflux from Macrophage Foam Cells via Lysosomal Acid Lipase. *Cell Metab* 13 655–667. 10.1016/j.cmet.2011.03.023 21641547 PMC3257518

[B37] Pasparakis M. Vandenabeele P. (2015). Necroptosis and its role in inflammation. *Nature* 517 311–320. 10.1038/nature14191 25592536

[B38] Ruan Q. Z. Rinkinen J. R. Doval A. F. Scott B. B. Tobias A. M. Lin S. J. (2019). Safety Profiles of Fat Processing Techniques in Autologous Fat Transfer for Breast Reconstruction. *Plast Reconstr Surg* 143 985–991. 10.1097/prs.0000000000005424 30921112

[B39] Sauler M. Bazan I. S. Lee P. J. (2019). Cell Death in the Lung: The Apoptosis-Necroptosis Axis. *Annu Rev Physiol* 81 375–402. 10.1146/annurev-physiol-020518-114320 30485762 PMC6598441

[B40] Schott M. B. Weller S. G. Schulze R. J. Krueger E. W. Mcniven M. A. (2019). Lipid droplet size directs lipolysis and lipophagy catabolism in hepatocytes. *J Cell Biol* 218 3320–3335. 10.1083/jcb.201803153 31391210 PMC6781454

[B41] Sergin I. Evans T. D. Razani B. (2015). Degradation and beyond: the macrophage lysosome as a nexus for nutrient sensing and processing in atherosclerosis. *Curr Opin Lipidol* 26 394–404. 10.1097/MOL.0000000000000213 26241101 PMC5027838

[B42] Sharma D. Kanneganti T. D. (2016). The cell biology of inflammasomes: Mechanisms of inflammasome activation and regulation. *J Cell Biol* 213 617–629. 10.1083/jcb.201602089 27325789 PMC4915194

[B43] Shi Y. Huang C. Yi H. Cao Q. Zhao Y. Chen J. (2020). RIPK3 blockade attenuates kidney fibrosis in a folic acid model of renal injury. *Faseb j* 10 10458. 10.1096/fj.201902544RR 32542792

[B44] Slack G. C. Tabit C. J. Allam K. A. Kawamoto H. K. Bradley J. P. (2014). Parry-Romberg reconstruction: beneficial results despite poorer fat take. *Ann Plast Surg* 73 307–310. 10.1097/SAP.0b013e31827aeb0d 23676519

[B45] Spencer M. Yao-Borengasser A. Unal R. Rasouli N. Gurley C. M. Zhu B. (2010). Adipose tissue macrophages in insulin-resistant subjects are associated with collagen VI and fibrosis and demonstrate alternative activation. *Am J Physiol Endocrinol Metab* 299 E1016–E1027. 10.1152/ajpendo.00329.2010 20841504 PMC3006260

[B46] Sun K. Park J. Gupta O. T. Holland W. L. Auerbach P. Zhang N. (2014). Endotrophin triggers adipose tissue fibrosis and metabolic dysfunction. *Nat Commun* 5 3485. 10.1038/ncomms4485 24647224 PMC4076823

[B47] Tanaka M. Ikeda K. Suganami T. Komiya C. Ochi K. Shirakawa I. (2014). Macrophage-inducible C-type lectin underlies obesity-induced adipose tissue fibrosis. *Nat Commun* 5 4982. 10.1038/ncomms5982 25236782

[B48] Thorp E. Subramanian M. Tabas I. (2011). The role of macrophages and dendritic cells in the clearance of apoptotic cells in advanced atherosclerosis. *Eur J Immunol* 41 2515–2518. 10.1002/eji.201141719 21952808 PMC3289088

[B49] Tian F. Yao J. Yan M. Sun X. Wang W. Gao W. (2016). 5-Aminolevulinic Acid-Mediated Sonodynamic Therapy Inhibits RIPK1/RIPK3-Dependent Necroptosis in THP-1-Derived Foam Cells. *Sci Rep* 6 21992. 10.1038/srep21992 26911899 PMC4766406

[B50] Tonnus W. Meyer C. Paliege A. Belavgeni A. von Mässenhausen A. Bornstein S. R. (2019). The pathological features of regulated necrosis. *J Pathol* 247 697–707. 10.1002/path.5248 30714148

[B51] Turner N. A. Mughal R. S. Warburton P. O’Regan D. J. Ball S. G. Porter K. E. (2007). Mechanism of TNFα-induced IL-1α, IL-1β and IL-6 expression in human cardiac fibroblasts: Effects of statins and thiazolidinediones. *Cardiovasc Res* 76 81–90. 10.1016/j.cardiores.2007.06.003 17612514

[B52] Vandenabeele P. Galluzzi L. Vanden Berghe T. Kroemer G. (2010). Molecular mechanisms of necroptosis: an ordered cellular explosion. *Nat Rev Mol Cell Biol* 11 700–714. 10.1038/nrm2970 20823910

[B53] Vila I. K. Badin P. M. Marques M. A. Monbrun L. Lefort C. Mir L. (2014). Immune cell Toll-like receptor 4 mediates the development of obesity- and endotoxemia-associated adipose tissue fibrosis. *Cell Rep* 7 1116–1129. 10.1016/j.celrep.2014.03.062 24794440

[B54] Webb N. R. Moore K. J. (2007). Macrophage-derived foam cells in atherosclerosis: lessons from murine models and implications for therapy. *Curr Drug Targets* 8 1249–1263. 10.2174/138945007783220597 18220702

[B55] Xiao X. Du C. Yan Z. Shi Y. Duan H. Ren Y. (2017). Inhibition of Necroptosis Attenuates Kidney Inflammation and Interstitial Fibrosis Induced By Unilateral Ureteral Obstruction. *Am J Nephrol* 46 131–138. 10.1159/000478746 28723681

[B56] Zhang D. W. Shao J. Lin J. Zhang N. Lu B. J. Lin S. C. (2009). RIP3, an energy metabolism regulator that switches TNF-induced cell death from apoptosis to necrosis. *Science* 325 332–336. 10.1126/science.1172308 19498109

[B57] Zhu K. Liang W. Ma Z. Xu D. Cao S. Lu X. (2018). Necroptosis promotes cell-autonomous activation of proinflammatory cytokine gene expression. *Cell Death Dis* 9 500. 10.1038/s41419-018-0524-y 29703889 PMC5923285

