# Supplementary material for: Necroptosis in Macrophage Foam Cells Promotes Fat Graft Fibrosis in Mice
Source: Front Cell Dev Biol. 2021 Mar 25;9:651360. doi: 10.3389/fcell.2021.651360 (PMC8027326; doi:10.3389/fcell.2021.651360)
Supplement: Supplementary file 1 [file Data_Sheet_1.docx]

***Supplementary Material***


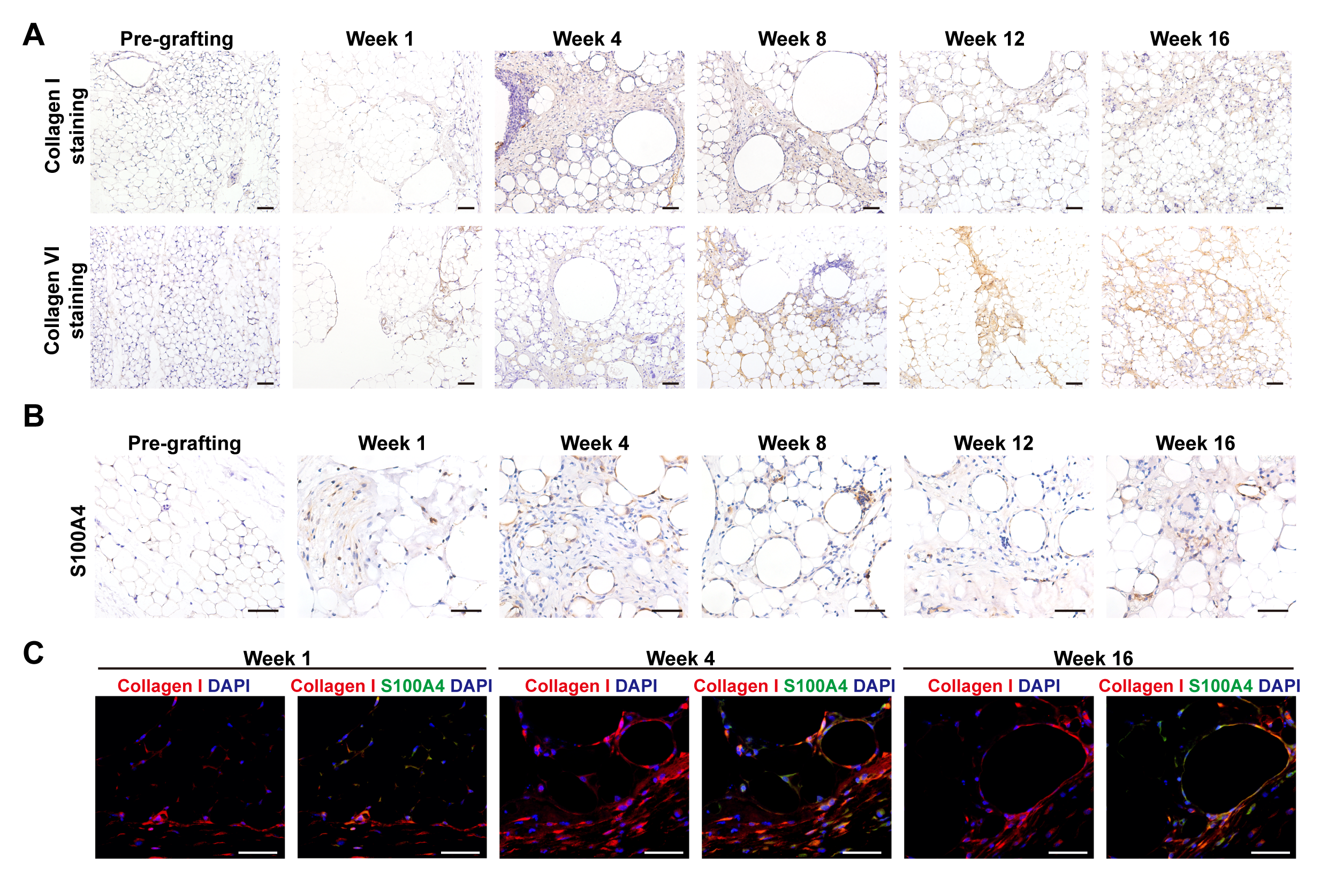


**Supplementary Figure 1 Collagen deposition is increased in fat graft tissue after grafting**

(A) Representative collagen I and collagen VI staining in fat tissue before and after grafting. (B) Representative S100A4 staining in fat tissue before and after grafting. (C) Representative immunofluorescence staining of collagen I (red) and S100A4 (green) in fat tissue before and after grafting. Scale bars = 50 μm.


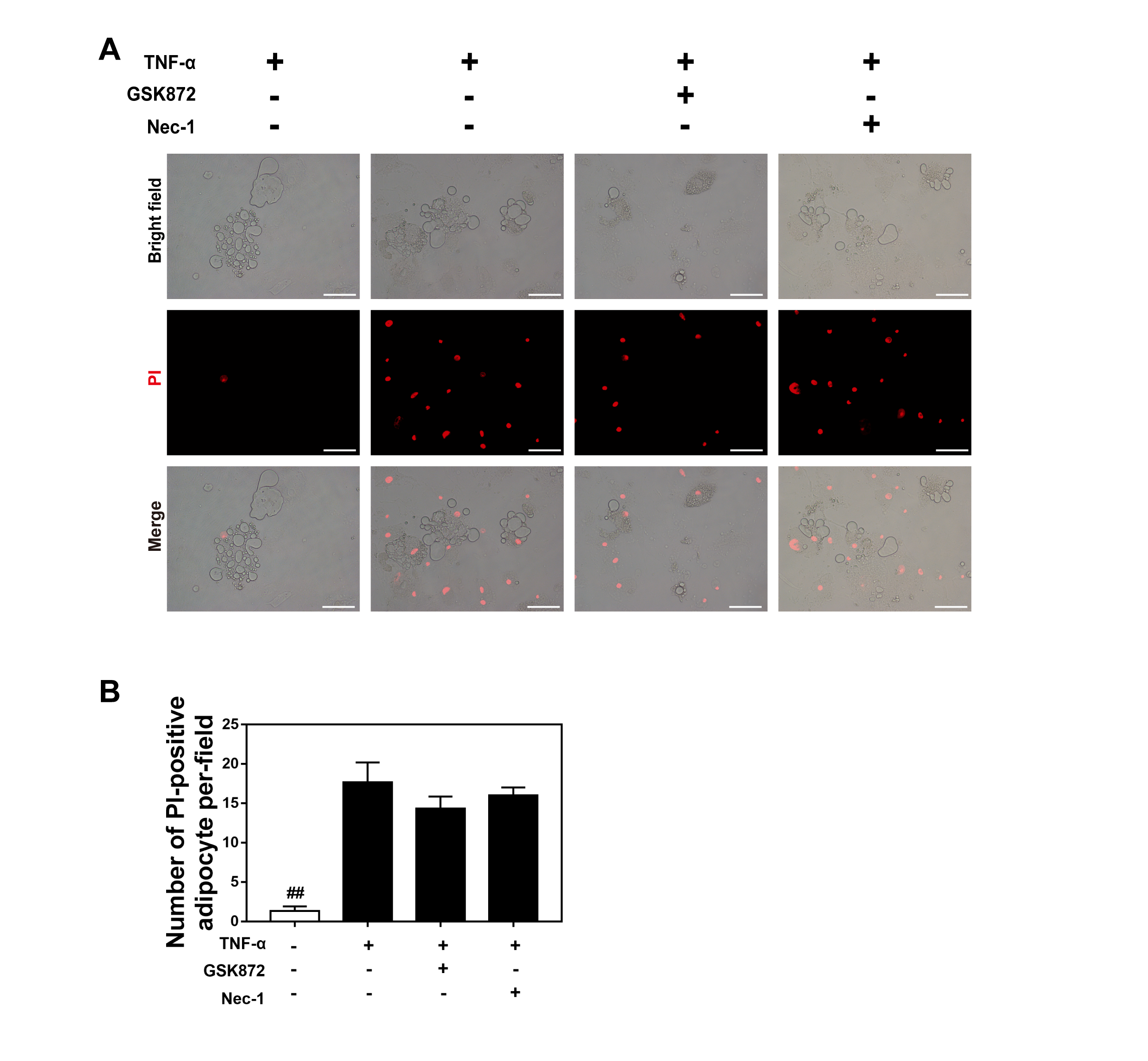


**Supplementary Figure 2 TNF-α was sufficient to induce apoptosis but not necroptosis in adipocytes *in vitro*.**

(A) Adipocytes were co-cultured with TNF-α in the presence or absence of a necroptosis inhibitor (Nec-1 or GSK872) for 24 h, and uninduced adipocytes were used as controls. PI staining was used to detect the cell necrosis of adipocytes in the four groups. (B) Quantification of the number of PI+ adipocytes showed that necroptosis inhibitors do not reduce the number of PI-positive cell in the *in vitro* apoptotic adipocyte model. Scale bars = 50 μm. Data are presented as the mean ± SD. ^##^*P* < 0.01 compared with the group of adipocytes co-cultured with TNF-α in the absence of necroptosis inhibitors.


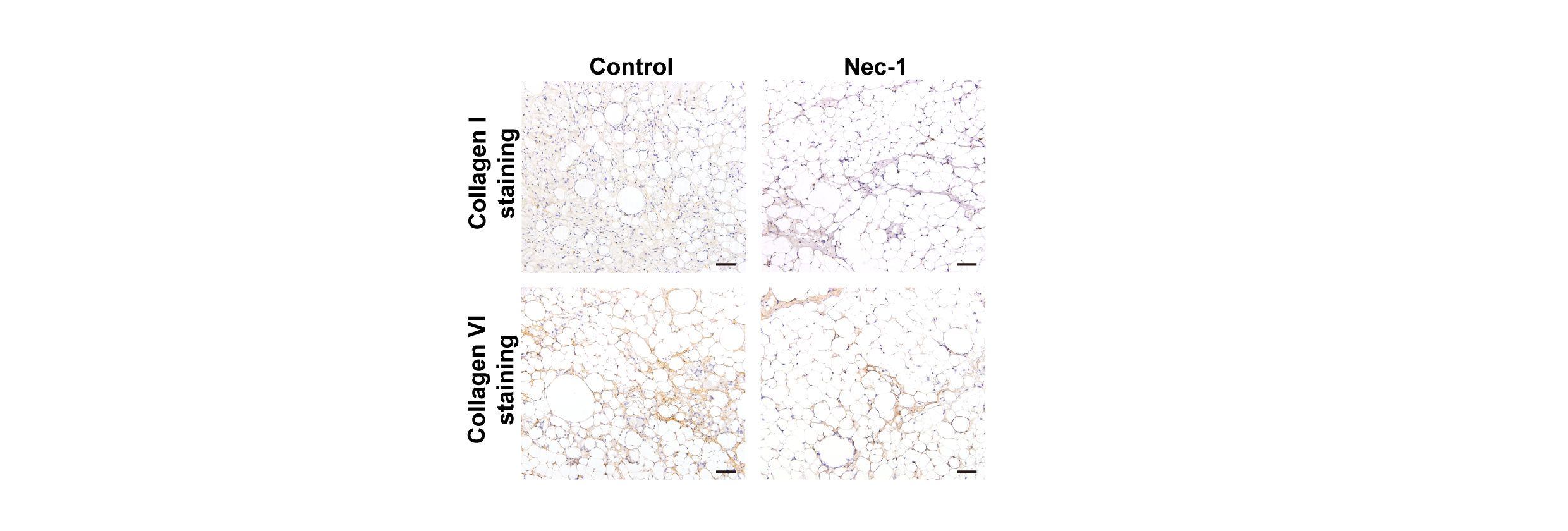


**Supplementary Figure 3 Treatment with Nec-1 alleviates collagen deposition in fat graft tissue**

Collagen I and collagen ng in fat tissue at week 16 in mice administered vehicle (control) or Nec-1. Scale bars = 50 μm.
